# Long-Term Oncological and Functional Outcomes after Laparoscopic Partial Nephrectomy with Hyperselective Embolization of Tumor Vessels in a Hybrid Operating Room

**DOI:** 10.3390/jcm12165167

**Published:** 2023-08-08

**Authors:** Ulysse Frantz, Antoine Bouvier, Thibaut Culty, Merzouka Zidane, Souhil Lebdai, Pierre Bigot

**Affiliations:** 1 Department of Urology, Angers University Hospital, 49000 Angers, France; thculty@chu-angers.fr (T.C.); solebdai@chu-angers.fr (S.L.); pibigot@chu-angers.fr (P.B.); 2 Department of Radiology, Angers University Hospital, 49000 Angers, France; anbouvier@chu-angers.fr; 3 Department of Pathological Anatomy and Cytology, Angers University Hospital, 49000 Angers, France; mmzidame@chu-angers.fr

**Keywords:** hybrid operating room, kidney cancer, laparoscopy, oncology, surgery

## Abstract

Laparoscopic partial nephrectomy (LPN) after hyperselective embolization of tumor vessels (HETV) in a hybrid operating room (HOR) that combines traditional surgical equipment with advanced imaging technology, is a non-clamping surgical approach to treat localized kidney tumors that has shown promising short-term results. The aim of this study was to evaluate the long-term oncological and functional outcomes of this procedure. All consecutive patients treated for a localized kidney tumor by LPN after HETV between May 2015 and October 2022 in a single academic institution were included in the study. Clinical, pathological and biological data were collected prospectively in the uroCCR database. We evaluated intraoperative data, postoperative complications, surgical margin and modification of renal function after surgery. We included 245 patients. The median tumor size was 3.2 (2.5–4.4) cm. The R.E.N.A.L. complexity was low, medium and high for 104 (43.5%), 109 (45.6%) and 26 (10.9%) patients, respectively. Median LPN time was 75 (65–100) min and median blood loss was 100 (50–300) mL. Surgical postoperative complications occurred in 56 (22.9%) patients with 17 (5.7%) major complications. The median Glomerular Function Rate variation at 6 months was −7.5 (−15–−2) mL/min. Malignant tumors were present in 211 (86.1%) patients, and 12 (4.9%) patients had positive surgical margins. After a median follow-up of 27 (8–49) months, 20 (8.2%) patients had a tumor recurrence and 4 (1.6%) died from cancer. At 5 years, disease free survival, cancer specific survival and overall survival rates were 84%, 96.8% and 88.3%, respectively. Performing LPN after HETV in a HOR is a safe and efficient non-clamping approach to treat localized kidney tumors.

## 1. Introduction

Kidney cancer is one of the most common types of cancer with an estimated 138,600 new cases and 54,000 deaths in Europe in 2020 [1]. International guidelines consider partial nephrectomy (PN) as the gold standard technique for localized kidney tumor treatment [2,3]. Different approaches are described, such as open partial nephrectomy (OPN), laparoscopic partial nephrectomy (LPN) or robot-assisted laparoscopic partial nephrectomy (RALPN). Compared to both OPN and LPN, RALPN provides decreased intraoperative blood loss, shorter hospitalization time, fewer complications and shorter ischemia times [4,5]. As a result, RALPN has become the new gold standard technique for mini-invasive PN [6]. LPN can be complex, with a prolonged learning curve, due to limited ergonomics and to technical challenges such as laparoscopic suture [7]. Gallucci et al. have described a zero-ischemia technique of PN after selective embolization of tumor vessels. The main disadvantages of their technique were prolonged delays between the embolization step and the surgical step (up to 24 h), resulting in peri-lesioned edema increasing the tumor dissection difficulty [8,9,10]. Next-generation hybrid operating rooms combine traditional surgical equipment with advanced imaging technology and allow both procedures to be performed together while optimizing the time between the embolization and the surgical steps [11]. LPN after hyperselective embolization of tumor vessels (HETV) in a hybrid operating room (HOR) is a non-clamping approach which has been performed in our center since 2015 [12]. The first short-term results were encouraging regarding operative times, bleeding, postoperative renal function, and the risk of arterial pseudoaneurysm [13,14,15]. The aim of this study was to evaluate the long-term oncological and functional results of LPN after HETV in HOR for localized kidney tumors.

## 2. Materials and Methods

### 2.1. Patients

We included all the patients treated for a localized kidney tumor by LPN after HETV in an HOR between May 2015 and October 2022 in our academic institution. Clinical, pathological, and biological data were collected prospectively, after informed consent, in the uroCCR database (NCT03293563, CNIL authorization number: DR-2013-206). The patient demographics studied were age, gender, BMI, solitary kidney, and ASA score. The indication of partial nephrectomy was imperative if patients had bilateral tumors, solitary kidney or chronic kidney disease (eGFR according to CKD-EPI < 60 mL/min/1.73 m^2^). CKD-EPI was used to assess pre- and postoperative renal functions. Tumor characteristics were side and size, histology, T stage and ISUP grade [16]. R.E.N.A.L. score was used to classify lesions into low (4–6), medium (7–9) and high (≥10) surgical complexity [17].

### 2.2. Intraoperative and Postoperative Outcomes

We evaluated the complete operating time (embolization plus laparoscopy) and the laparoscopic time alone, blood loss, the number of arterial branches embolized, intraoperative complications and transfusions. We rated postoperative complications, occurring within the first 30 days, according to the Clavien–Dindo classification [18]. Major postoperative complications were defined as Clavien score >2. We analyzed the length of hospital stay, surgical margins and oncological recurrences. The need for complementary local intervention such as new partial nephrectomy, radical nephrectomy or radiofrequency was assessed in patients with malignant pathology.

### 2.3. Angiographic and Surgical Procedure

**Figure 1:**
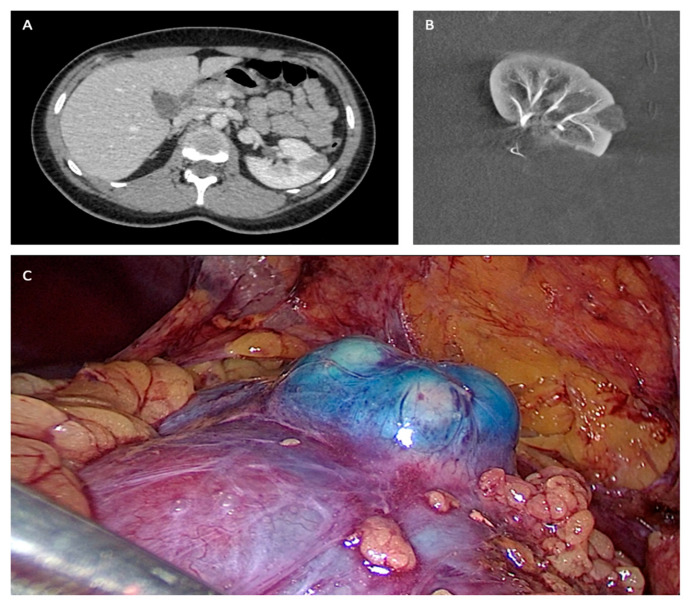
(**A**) Preoperative TDM. (**B**) Post embolization angiography. (**C**) Per-operative laparoscopic view.

All procedures were performed under general anesthesia in a Discovery IGS 730 (GE Healthcare) operating room by different urological surgeons (*n* = 15) and interventional radiologists (*n* = 3). First, patients were positioned in a standard decubitus position. The interventional radiologist inserted a 4F introducer in the opposite side femoral artery to the operate tumor. Endovascular navigation was facilitated with the superimposition of the preoperative CT scan on live fluoroscopy. A 3D renal arteriography was performed to precisely identify the tumor vascularization. Superselective catheterization of the subsegmental artery feeding the tumor was achieved with a coaxial microcatheter. The endovascular guidance software Flight Plan was used in order to highlight vessels traveling to the tumor. Highly selective embolization was made in order to spare as much renal tissue as possible, with a 250 mm calibrated Embozene microsphere (CeloNova Bioscience Inc., San Antonio, TX, USA), fibered microcoils (Vortex, 2 and 3 mm diameter, Boston Scientific, Marlborough, MA, USA) or 0.2–0.3 mL of glue N-butyl-2- yanoacrylate Glubran 2 (GEM Srl, Viareggio, Italy) diluted 1:5 with Lipiodol (Guerbet, Paris, France). In order to facilitate the tumor localization by the surgeon during the laparoscopy, the interventional radiologist realized, immediately before the embolization, a superselective injection into tumoral arteries of a blue dye (Patente Blue V, Guerbet, Paris, France) in order to color the tumor. At the end of the embolization procedure, a 3D arteriography was realized to control the complete occlusion of the tumor vessels. The introducer was kept in position to keep vascular access for a potential control of bleeding during the laparoscopic procedure with an endovascular balloon or a second embolization. Then, patients were positioned in a lateral decubitus position in order to perform standard LPN. The LPN procedure was performed without individuating the renal pedicle, and the tumor was enucleated [12,19] (Figure 1).

### 2.4. Follow-Up

Patients had postoperative visits at one month and six months. In case of malignant tumor, follow-up rhythm was defined according to the French oncology recommendation guidelines [2]. Surveillance was conducted using a CT-scan as well as physical and biological analysis.

### 2.5. Statistical Analysis

Statistical analysis was conducted according to guidelines. Disease-free survival (DFS), cancer-specific survival (CSS) and overall survival (OS) were calculated based on the Kaplan–Meier method. Recurrence risk factors were evaluated in patients with malignant pathology using a Cox regression analysis. We analyzed complication risk factors and risk factors for significant GFR decrease (>−10 mL/min) by conducting univariate analysis and multivariate analysis. The significance threshold, *p,* was set at 0.05.

Qualitative variables were described with absolute values and percentages, and quantitative values were described with median, first quartile and third quartile. Statistical analyses were performed with SPSS^®^ Version 15.0 Software (IBM Corp., Armonk, NY, USA).

## 3. Results

### 3.1. Patient and Tumor Characteristics

Between May 2015 and October 2022, 245 patients were included in this study. There were 163 (66.5%) males and 82 (33.5%) females. The median age was 64 (52–72) years. The operative indication was elective for 215 (87.8%) patients and imperative for 30 (12.2%) patients. The median tumor size was 3.2 (2.5–4.4) cm. The R.E.N.A.L. complexity was low, moderate and high for 104 (43.5%), 109 (45.6%) and 26 (10.9%) patients, respectively. Patient and tumor characteristics are reported in Table 1.

### 3.2. Operative Outcomes

Median total operative and laparoscopic times were, respectively, 168 (145–199) min and 75 (60–100) min. The number of arterial branches embolized was 1, 2, 3, 4, and 5 for 88 (36.1%), 99 (40.6%), 41 (16.8%), 15 (6.1%) and 1 (0.4%) patient, respectively. The median blood loss was 100 (50–300) mL. Only one clamping of the renal pedicle was necessary. The tumor was located in the middle of the kidney and was completely endophytic, with a R.E.N.A.L. nephrometry score of 12. For eight (3.3%) patients, cortical suturing was needed.

There were 10 intraoperative complications (4.1%): 5 hemorrhages with transfusion required in 4 (1.6%) patients, 2 vascular wounds that were clipped, 1 splenic wound that was coagulated, 1 ureteral wound that required intraoperative suture and a JJ catheter and 1 open conversion for tumor localization difficulties. Intraoperative data are reported in Table 2.

### 3.3. Postoperative Outcomes

The median hospital stay was 4 (3–4) days. A total of 56 postoperative complications occurred with 39 (16%) minor and 17 (6.9%) major complications. Post-operative outcomes are reported in Table 2. We did not identify a predictive factor of major postoperative complications (Table 3).

### 3.4. Oncological Outcomes

There were 211 (86.1%) malignant tumors and 34 (13.9%) benign tumors. For the malignant tumors the pT stage was 1a, 1b, 2a, 2b and 3a in 142 (67.3%), 42 (19.9%), 9 (4.3%), 1 (0.5%) and 17 (8.1%) patients, respectively. Surgical margins were positive in 12 (4.9%) patients. There were 20 (9.5%) tumor recurrences with 17 (8.1%) local recurrences and 7 (3.3%) metastatic progressions. Local recurrences were treated by radical nephrectomy in eight (3.8%) patients, radiofrequency ablation in six (2.8%) patients, intraperitoneal nodule excision in two (1%) patients, and by a new partial nephrectomy in one (0.5%) patient. During a median follow-up of 27 (8–49) months, 14 (5.7%) patients died with 4 (1.6%) specific deaths. Oncological outcomes are reported in Table 4.

In multivariable analysis, T stage (HR:4.4, *p* = 0.027) and surgical margins (HR:4.29, *p* = 0.029) were identified as recurrence risk factors (Table 5).

**Figure 2:**
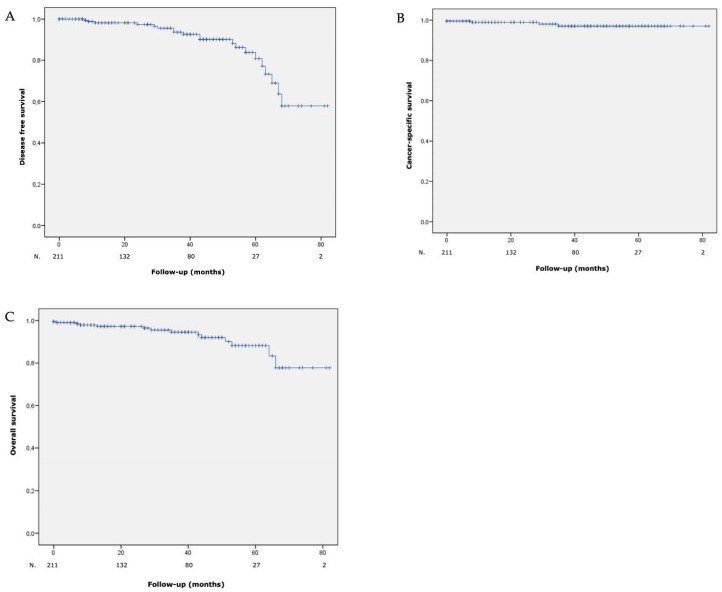
(**A**) Disease free survival, (**B**) cancer-specific survival and (**C**) overall survival after laparoscopic partial nephrectomy with hyperselective embolization of tumor vessels in a hybrid operating room for 211 malignant renal tumors.

At 5 years, DFS, CSS and OS rates were 84%, 96.8% and 88.3%, respectively. Curves are shown in Figure 2.

### 3.5. Functional Outcomes

**Figure 3:**
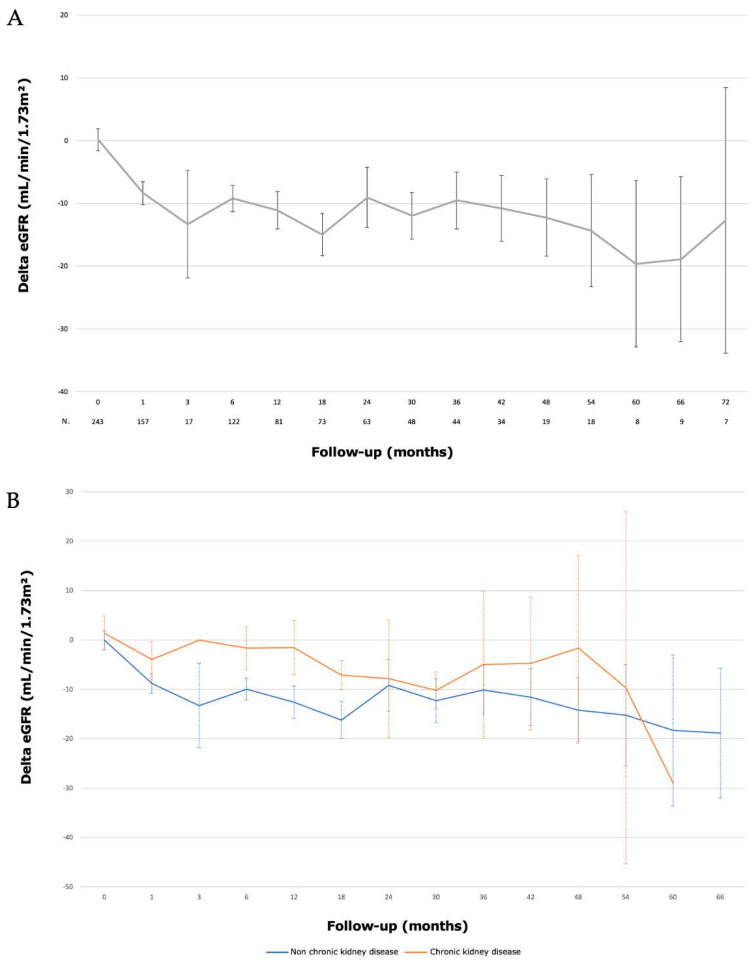
Evolution of renal function (eGFR) after laparoscopic partial nephrectomy with hyperselective embolization of tumor vessels in a hybrid operating room for (**A**) 243 localized renal tumors and (**B**) according to pre-existant chronic kidney disease (eGFR < 60 mL/min).

The median preoperative eGFR (CKD-EPI) was 90.5 (77–101.8) mL/min/1.73 m^2^. The median eGFR (CKD-EPI) at one month and six months were 82 (67.3–93.8) mL/min/1.73 m^2^ and 82 (66–95) mL/min/1.73 m^2^, respectively. The median eGFR (CKD-EPI) at 1, 2, 3, 4 and 5 years were 77 (70.6–81.1) mL/min/1.73 m^2^; 78.5 (70.6–82.7) mL/min/1.73 m^2^; 79.5 (70.5–85) mL/min/1.73 m^2^; 72.5 (59–80.6) mL/min/1.73 m^2^ and 85 (44.3–99.7) mL/min/1.73 m^2^, respectively. Evolution of renal function is described in Figure 3. At the end of the follow-up, a significant decrease in GFR (>−10 mL/min) was present in 107 (43%) patients. We did not identify any risk factor for a significant decrease in renal function (Table 6).

## 4. Discussion

In this study, we reported a series of LPN with HETV in HOR with a median follow-up of 27 months. The demographic data of our study were similar to those already published, in terms of age, BMI, ASA scores, tumor size, and R.E.N.A.L. complexity [4,5,6,20].

The median total operative time of 168 min, including HETV, patient repositioning and LPN, appears to be shorter compared to other LPN series [6,21]. Our operative times seem similar to those described in the RALPN series [4,5]. The median laparoscopic time alone was reduced to 75 min. This could be explained by the absence of renal pedicle dissection and the possibility of suturless PN in most of the cases. In addition, blue dye embolization facilitated macroscopic tumor localization without the use of intra-body ultrasound [19]. In our study, only one (0.4%) laparoconversion was necessary due to the difficulty in tumor localization. Simmons et al. described six (1%) cases of conversion [22], while Masson-Lecomte et al. had seven (3.18%) conversions in the robot-assisted laparoscopy group and five (11.1%) in the laparoscopy group [23]. Dissection was always possible without difficulty related to perilesional edema; a limiting factor described when embolization was performed remotely from the surgical procedure [10].

Intraoperative bleeding is the most important and severe complication of PN. In contemporary RALPN series, bleeding occurred in 6% of the cases. Despite the off-clamp PN, intraoperative bleeding was 100 mL which is lower than bleeding reported in RALPN (150 to 300 mL) [4,5,24]. Hemostasis was achieved using hemostatic agents and in the majority of the cases did not require cortical suturing (96.7%), which could contribute to the preservation of healthy renal parenchyma [25]. Furthermore, only five (2.1%) major hemorrhages occurred, and four of them required intraoperative blood transfusions. These results are lower than those reported in the literature [4,6,21,26].

The low rate of intraoperative hemorrhagic complications, preventing organ hypoperfusion, could also contribute to nephron preservation. However, we did have three (1.2%) postoperative renal bleeding episodes and one (0.4%) hepatic bleeding episode, which required additional postoperative embolization. For one patient the bleeding occurred because of an embolization failure. For two other patients, bleeding occurred because of a large tumor excision with non-embolized healthy renal parenchyma removal. Simone et al. described two cases (1%) of secondary bleeding requiring additional embolization in their series of LPN after HETV [9]. However, these complications remain lower than those reported by George et al. who performed 16 (5.54%) embolizations for postoperative bleeding [26]. The use of glue for embolization appears to be a safe and effective technique [15].

Major postoperative complications were noted in 17 (6.9%) patients, which is consistent with the literature [6]. One patient died postoperatively from cardiac arrest secondary to uncontrolled asthma and had a history of multiple cardiovascular comorbidities (high blood pressure and aortic valve disease with mechanical cardiac valve). Minor complications were mostly isolated postoperative hyperthermia, related to post-embolization syndrome [14]. Hospitalization times were similar to those reported in RALPN trials [20].

The deterioration of renal function after PN is a multifactorial and complex process related to non-modifiable factors (age, comorbidities and preoperative kidney function) and modifiable factors (duration of ischemia and nephron sacrifice) [7]. Mir et al. described a preservation of approximately 90% of renal function after PN [27].

In a previous study, we evaluated renal function by GFR and computed tomography renal volume 6 months after surgery in 137 patients [15]. We found a 9.3 mL/min decreased in GFR and a median loss of 21 mL of healthy parenchyma on the operated kidney which is consistent with the literature on robot-assisted surgery [28]. With a larger population and a longer follow-up, we found a 10% loss of renal function, which remained stable over time. We did not identify the risk factor of significant decrease in renal function. However, we did not have data regarding the volume of healthy renal parenchyma loss nor any pre-existing hypertension or proteinuria. This could be a limitation as it appears that these factors may have an impact on the postoperative GFR [29,30,31].

Preoperative embolization of tumor arteries has several advantages: (1) the reduction in intraoperative bleeding; (2) the selective dissection of the tumor using blue coloration of the tumor to optimize the differentiation from the normal parenchyma and therefore resulting in a better preservation; and (3) performing PN without clamping which avoids the risk of renal ischemia lasting more than 25 to 30 min [32]. This is an important point especially for patients suffering from preoperative chronic renal disease: HETV limited the loss of renal function to 9% at 42 months. These results should be interpreted with caution, as our population of chronic renal insufficiency patients was only 30 patients and only 4 patients were still followed-up at 42 months. Nevertheless, studies specifically focusing on this population would be interesting.

Out of the 245 patients in our series, 34 (13.9%) had benign tumors. These results are consistent with the literature. Simone et al. found 30% benign tumors, Masson-Lecomte et al. had 16% in the robot-assisted series, and Peyronnet et al. had 14.6% [4,9,22,23]. Of these 34 benign tumors, 22 were oncocytomas. However, the diagnosis is difficult, and it can be tricky to identify chromophobe renal cell carcinoma, with 9 to 25% of patients having a final diagnosis of clear cell carcinoma [33,34]. The other benign tumors included five angiomyolipomas, five symptomatic cysts, one hemangioma, and a metanephric adenoma.

We identified two risk factors for recurrence: positive surgical margins and pathological stage ≥ pT3a. The rate of positive margins was 4.9%. These results are similar to the robot-assisted series of Ingels et al. (4.9%), Peyronnet et al. (5.2%), Pignot et al. (5.7%) and Masson-Lecomte et al. (8%) [4,5,20,23]. Despite a positive surgical margin rate comparable to that observed in the literature, the 5-year disease-free survival rate in our series (84%) appears to be in the low range compared to multicenter series, which reported that 5-year CFS estimate rates ranged from 86.4% to 98.4% [35]. It is probably related to the aggressiveness and complexity of the tumors treated in our series. Indeed, 8.1% of our patients had a pT3a tumor with invasion of the peri-renal fat. There were also two atypical histologies in our series (metastasis from lung cancer and Bellini’s carcinoma) which recurred in the year following surgery. Moreover, most series reporting long-term oncological results are retrospective studies whereas our data were collected prospectively, and this may constitute a bias. For example, two recent prospective studies reported 5-year CFS estimate rates of 86% and 91%, which is closer to what we report [36,37].

All studies comparing RALPN to OPN or LPN have found benefits for the robot-assisted approach regarding postoperative complications, bleeding, transfusions rate and length of hospital stay. However, our results appear encouraging regarding the robot-assisted LPN series [4,5,6,23].

The costs associated with the use of robot-assisted surgery are expensive. It could be interesting to compare them to those related to the use of an HOR.

Our study is of course not without limitations. It is an observational study, with no control group and the different comparisons with other surgical approaches, particularly robot-assisted, may be debatable. No conclusion can be drawn about the superiority of one technique over another.

However, the main strength of our study is being in a real-life prospective setting. It represents the activity of our academic department, and the surgical procedures were performed by operators with different levels of experience, some of whom were just beginning their learning curve in laparoscopy.

## 5. Conclusions

Long-term oncological and functional outcomes show that laparoscopic partial nephrectomy after hyperselective embolization of tumor vessels in a hybrid operative room is a feasible, reproducible and safe approach to treat localized kidney tumors. Further prospective studies should be carried out to confirm these results.

## Figures and Tables

**Table 1:** Patients and tumors characteristics.

	Laparoscopic Partial Nephrectomy after Hyperselective Embolization of Tumor Vessels (*n* = 245)
Median age, years [IQR]	64 [52–72]
Gender, *n* (%)	
Male	163 (66.5)
Female	82 (33.5)
Median BMI, kg/m^2^ [IQR]	27.3 [24.5–30.8]
Solitary kidney, *n* (%)	2 (0.8)
ASA score, *n* (%)	
ASA 1	43 (17.6)
ASA 2	145 (59.4)
ASA 3	55 (22.5)
ASA 4	1 (0.5)
Side, *n* (%)	
Right	127 (51.8)
Left	118 (48.2)
Median tumor size, cm [IQR]	3.2 [2.5–4.4]
R.E.N.A.L. complexity, *n* (%)	
Low	104 (43.5)
Moderate	109 (45.6)
High	26 (10.9)
Indication, *n* (%)	
Elective	215 (87.8)
Imperative	30 (12.2)
Median preoperative eGFR CKD-EPI, mL/min/1.73 m^2^ [IQR]	90.5 [77–101.8]
Median follow-up, month [IQR]	27 [8–49]

BMI = Body Mass Index; IQR = Inter-Quartile Range; and eGFR = estimated Glomerular Filtration Rate.

**Table 2:** Intraoperative and postoperative characteristics.

	Laparoscopic Partial Nephrectomy after Selective Embolization of Tumor Vessels (*n* = 245)
Median total operative time, min [IQR]	168 [145–199]
Median laparoscopic time, min [IQR]	75 [60–100]
Median blood loss, mL [IQR]	100 [50–300]
Number of arterial branches embolized, *n* (%)	
1	88 (36.1)
2	99 (40.6)
3	41 (16.8)
4	15 (6.1)
5	1 (0.4)
Intraoperative complications, *n* (%)	10 (4.1)
Intraoperative transfusions, *n* (%)	4 (1.6)
Postoperative complications CLAVIEN, *n* (%)	
I	32 (13.1)
II	7 (2.9)
IIIa	3 (1.2)
IIIb	12 (4.9)
IVa	1 (0.4)
V	1 (0.4)
Median hospital stay, days [IQR]	4 [3–4]

IQR: Inter-Quartile Range.

**Table 3:** Post-operative complications according to the Clavien–Dindo Classification.

	All Tumors (*n* = 245)	Low Complexity Group 1 (*n* = 104)	Moderate Complexity Group 2 (*n* = 109)	High Complexity Group 3 (*n* = 26)	*p* Value
All complications, *n* (%)	56 (22.9)	24 (23)	23 (21)	7 (26)	0.8
Major complications (Clavien > 2), *n* (%)	17 (6.9)	4 (3.8)	7 (6.4)	4 (15)	0.095

(1) R.E.N.A.L. Score 4–6: Low complexity. (2) R.E.N.A.L. Score 7–9: Moderate complexity. (3) R.E.N.A.L. Score 10–12: High complexity.

**Table 4:** Oncological outcomes.

	Laparoscopic Partial Nephrectomy after Selective Embolization of Tumor Vessels (*n* = 245)
Histology, *n* (%)	
Benign	34 (13.9)
Angiomyolipoma	5 (2)
Renal cyst	5 (2)
Oncocytoma	22 (9)
Hemangioma	1 (0.4)
Metanephric adenoma	1 (0.4)
Malignant	211 (86.1)
Clear cell renal cell carcinoma	158 (64.5)
Collecting duct/Bellini duct carcinoma	1 (0.4)
Chromophobe renal cell carcinoma	16 (6.5)
Papillary renal cell carcinoma	34 (13.9)
Eosinophilic renal cell carcinoma	1 (0.4)
Pulmonary metastasis	1 (0.4)
pT stage, *n* (%)	
pT1a	142 (67.3)
pT1b	42 (19.9)
pT2a	9 (4.3)
pT2b	1 (0.5)
pT3a	17 (8.1)
ISUP grade, *n* (%)	
1	22 (10.5)
2	125 (59.2)
3	40 (19)
4	6 (2.8)
NA	18 (8.5)
Surgical margins, *n* (%)	
Negative	233 (95.1)
Positive	12 (4.9)
Recurrences, *n* (%)	
All recurrences	20 (9.5)
Local recurrences	17 (8.1)
Metastatic progression	7 (3.3)
Surgical reoperation, *n* (%)	17 (8.1)
Totalisation	8 (3.8)
Partial nephrectomy	1 (0.5)
Radiofrequency ablation	6 (2.8)
Extra peritoneal nodule excision	2 (1)
Deaths, *n* (%)	14 (5.7)
Specific deaths, *n* (%)	4 (1.6)

**Table 5:** Risk factor of recurrences.

	Hazard Ratio (CI 95%)	*p* Value
Age, (continuous)	1.02 (0.977; 1.07)	0.340
Tumor size, (continuous)	1.15 (0.843; 1.55)	0.386
Indication NSS		
Elective	Reference	—
Imperative	1.00 (0.289; 3.47)	0.997
Histology		
Other	Reference	—
Clear cell renal cell carcinoma	1.77 (0.496; 6.33)	0.378
ISUP grade		
1/2	Reference	—
3/4	1.71 (0.618; 4.75)	0.300
T Stade		
T1 and T2	Reference	—
≥T3	4.04 (1.18; 13.91)	0.027
Surgical margins		
Negative	Reference	—
Positive	4.29 (1.17; 15.82)	0.029

Multivariate analysis with Cox proportion hazard regression.

**Table 6:** Risk factor for GFR decline > 10 mL/min.

	Hazard Ratio (CI 95%)	*p* Value
Gender (male vs. female)	0.78 (0.44; 1.3)	0.78
Age (continuous)	1.01 (0.99; 1.03)	0.33
Malignant tumor (vs. benign tumor)	0.75 (0.34; 1.6)	0.363
Tumor size (continuous)	0.97 (0.81; 1.16)	0.76
Preoperative chronic kidney disease (vs. GFR > 60 mL/min)	3.8 (0.79; 18.6)	0.095
Operative bleeding (continuous)	1 (0.99; 1.001)	0.48
Number of segmental arteries embolized > 1 (vs. 1)	0.99 (0.57; 1.71)	0.99

## Data Availability

Data presented are contained within the article; for additional information, datasets are also available upon request from the corresponding author.
